# Supplementary figures and images for: Application of recommended preventive measures against COVID-19 could help mitigate the risk of SARS-CoV-2 infection during dental practice: Results from a follow-up survey of French dentists
Source: PLoS One. 2021 Dec 22;16(12):e0261439. doi: 10.1371/journal.pone.0261439 (PMC8694455; doi:10.1371/journal.pone.0261439)

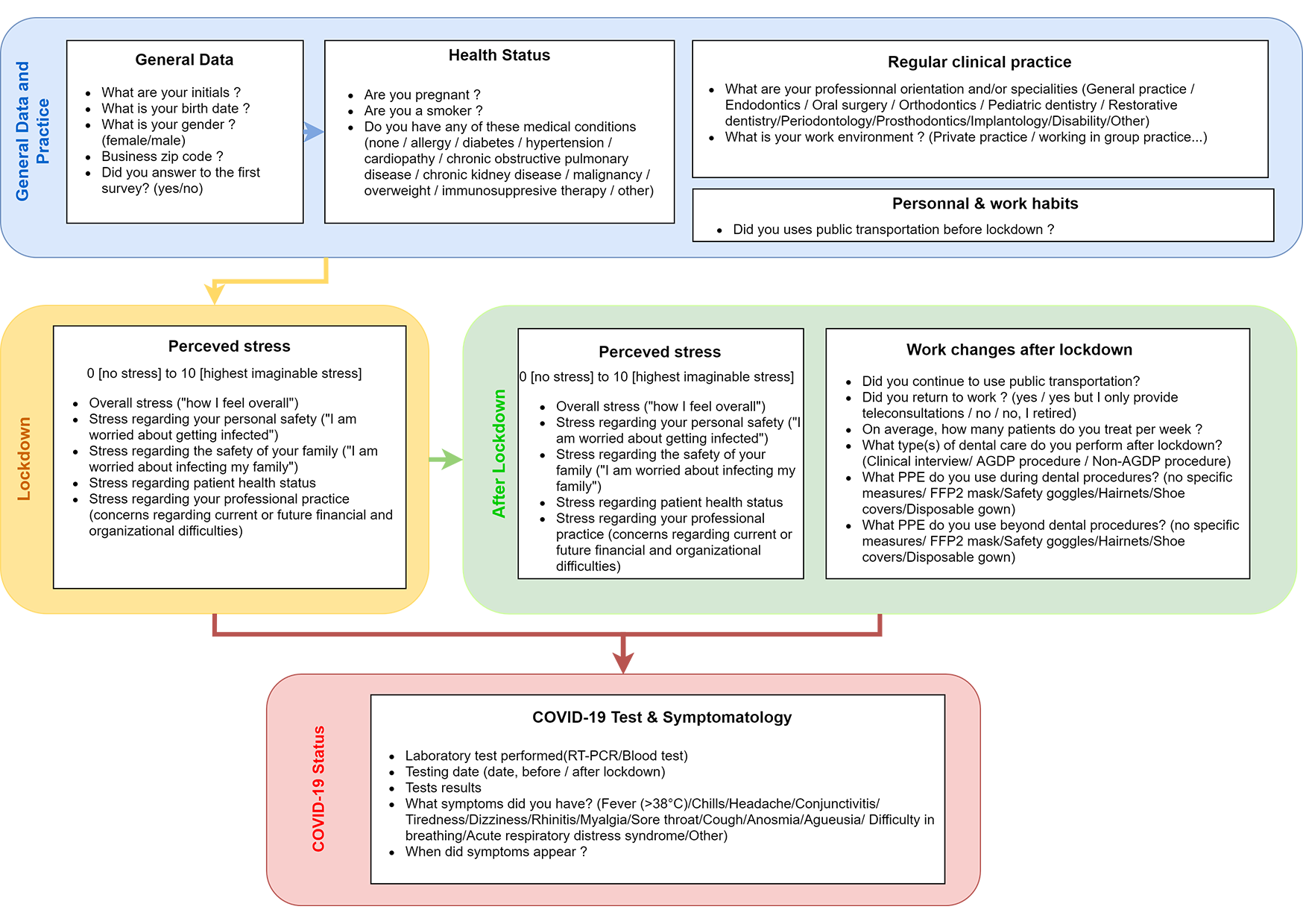

Supplement: S1 Fig — (TIF) [file pone.0261439.s001.tif]
